# Exposure to Perfluoroalkyl Substances During Pregnancy and Fetal BDNF Level: A Prospective Cohort Study

**DOI:** 10.3389/fendo.2021.653095

**Published:** 2021-06-01

**Authors:** Guoqi Yu, Fei Luo, Min Nian, Shuman Li, Bin Liu, Liping Feng, Jun Zhang

**Affiliations:** ^1^ Ministry of Education-Shanghai Key Laboratory of Children’s Environmental Health, School of Public Health, Shanghai Jiao-Tong University School of Medicine, Shanghai, China; ^2^ Ministry of Education-Shanghai Key Laboratory of Children’s Environmental Health, Xinhua Hospital, Shanghai Jiao-Tong University School of Medicine, Shanghai, China; ^3^ Department of Obstetrics and Gynecology, Duke University, Durham, NC, United States

**Keywords:** perfluoroalkyl substances, BDNF, gender difference, fetus, cohort

## Abstract

**Background:**

Humans are widely exposed to environmental perfluoroalkyl substances (PFAS), which may affect fetal neurodevelopment. Brain-derived neurotrophic factor (BDNF) is an important factor in neurodevelopment, but its role in PFAS-induced neurotoxicity is unclear. We investigated the association between prenatal PFAS exposure and fetal BDNF level in the umbilical cord blood in a large prospective cohort.

**Methods:**

A total of 725 pregnant women who participated in the Shanghai Birth Cohort were included. 10 PFAS were measured by high-performance liquid chromatography/tandem mass spectrometry (HPLC/MS-MS) in the plasma samples of early pregnancy. The BDNF level was determined by ELISA. The concentration of total mercury (Hg) in the umbilical cord blood was tested by cold vapor atomic absorption spectrometry (AAS) and included as a main confounder, along with other covariates. Multiple linear regression was used to explore the associations between PFAS concentrations and BDNF level. Quantile-based g-computation was applied to explore the joint and independent effects of PFAS on BDNF level.

**Results:**

The mean BDNF level in the total population was 10797 (±4713) pg/ml. Male fetuses had a higher level than female fetuses (*P*<0.001). A significant positive association was observed between PFHxS and BDNF level after adjusting for potential confounders [β=1285 (95% CI: 453, 2118, *P*=0.003)]. No association was observed between other PFAS congeners and BDNF level. Results of the mixed exposure model showed that the joint effects of PFAS mixture were not associated with BDNF [β=447 (95% CI: -83, 978, *P*=0.10)], while the positive association with PFHxS exposure remained significant after controlling for other PFAS [β=592 (95% CI: 226, 958, *P*=0.002)]. The above associations were more prominent in male [β=773 (95% CI: 25, 1520, *P*= 0.04)] than female fetuses [β=105 (95% CI: -791, 1002, *P*= 0.82)] for the mixed effects.

**Conclusions:**

Prenatal exposure to PFHxS was associated with an increased BDNF level in the umbilical blood, especially in male fetuses.

## Introduction

Per- and polyfluoroalkyl substances (PFAS) are a group of synthetic chemicals and have been used extensively in industrial and consumer products due to their thermal stability, hydrophobic and oleophobic properties. They are commonly used as additives in oil- and water-resistant paper, clothing, carpets, food packing material and kitchenware. Foods and drinking water are the major exposure sources for the general population. Even though perfluorooctane sulfonic acid (PFOS) and perfluorooctanoic acid (PFOA) had been phased out in industrial production in most western countries, they are still universally detectable across populations worldwide due to their long persistence and bioaccumulation in human biomonitoring (1). Moreover, the total production of PFAS (including traditional PFOA and PFOS and their alternatives) is still increasing rapidly in Asian countries, especially in China, due to the relocation of the production lines (2, 3). Even though PFAS have been linked to a variety of health outcomes, the toxicity of many PFAS remains unclear (4).

Fetuses are exposed to PFAS as these chemicals can cross the placental barrier actively or passively (5). Placental passage of PFAS depends on the carbon length, functional group (such as a sulfonate group), linear or branched isomers and their binding affinity to blood proteins like fatty acid binding protein (6). The potential effects of PFAS exposure on human neurodevelopment are gaining more attention. Previous epidemiologic studies showed that PFAS exposure may affect neurocognitive function. Prenatal exposure to PFAS was associated with the gross motor development domain at 2 years of age (7), childhood visual motor abilities (8, 9), IQ test scores in children at age 5 and 8 years (10, 11), externalizing behavioral difficulties (12), executive function at 8 years (13), and neuropsychological development at 4 years (14). PFAS exposure was also found to be positively associated with impulsivity and attention deficit hyperactivity disorder (ADHD) in school-aged children (15–17). Some of the studies further found that the effects of PFAS on neurodevelopment were sexually dimorphic (18, 19). However, findings to date are rather inconsistent and inconclusive. Several studies report protective or null associations between prenatal PFAS exposure and child neurobehavior (20–22). Further studies are needed.

BDNF is a cognitive-related protein mainly expressed in the central nervous system (including the cortex and hippocampus), and an important factor in the growth, development, maintenance and plasticity of the neurons (23). Moreover, BDNF plays a key role in maintaining the long-term potentiation (LTP) and long-term memory (LTM) of the hippocampus, contributing to learning and memory (24). BDNF could also play an essential role in supporting survival in a variety of neuronal populations in the central nervous system and protecting neurons against neurodegenerative disease and neuropsychological diseases, including Alzheimer’s disease and ADHD through activating MAPK/ERK, PI3K and TrkB mediated PLC-gamma pathways (25). Studies showed that prenatal exposure to a spectrum of polycyclic aromatic hydrocarbon (PAH)/aromatic pollutants may adversely affect early neurodevelopment, in part by reducing BDNF levels during the fetal period (26). A decreased BDNF level may be part of the biochemical basis of chronic arsenic exposure-related cognitive impairment (27). In addition, BDNF may also mediate the neurotoxic effects of polybrominated diphenyl ether congeners, dioxin, bisphenol A, nonylphenol, phthalate and particular matter exposure (28–31).

PFAS may affect synaptic plasticity, protein kinase C, calcium homeostasis, cellular differentiation or act *via* the thyroid hormone system and, eventually, exert its neurobehavioral effects (32–34). However, it is not clear whether BDNF plays a role in the neurotoxicity that PFAS may induce. *In vitro* experiments suggested that PFOS may inhibit BDNF-ERK-CREB signaling by increasing BDNF-related-microRNA levels, which may partially explain the mechanism of PFOS neurotoxicity (35, 36).

We aimed to examine the associations between PFAS levels in early pregnancy and fetal BDNF level in the umbilical cord blood from a large prospective cohort in China. The effects of the joint exposure and the independent effect of PFAS congeners and Hg were further explored. We also sought to evaluate whether the associations vary by child sex.

## Material and Methods

### Study Population

The present study was based on the longitudinal Shanghai Birth Cohort (SBC), which recruited women in early pregnancy from six participating hospitals in Shanghai, China, during 2013-2016. Detailed description of the cohort was provided previously (37). In brief, women were potentially eligible if they were at least 20 years old, registered Shanghai resident or married to a Shanghai resident; planned to seek prenatal care and give birth at the collaborative hospitals; were willing to sign a consent form and have follow-up visits regularly in the following 2 years. Participants provided the following information through an in-person interview: demographic characteristics (e.g. maternal age, race/ethnicity, height, pre-pregnancy weight, education, family economic status), health behavior and lifestyle (e.g. smoking and drinking status in early pregnancy, physical activities in early pregnancy) and reproductive history (e.g. parity), disease history (e.g. diabetes). After delivery, medical records including the delivery outcomes, birth outcomes, etc. were retrieved and reviewed by trained research staff to collect information on the pregnancy.

A total of 3527 pregnant women had PFAS measured, 820 had BDNF and 2131 had Hg measured. BDNF was not measured in some samples due to severe hemolysis. 725 mother-child pairs (21% of the cohort) were finally matched and included in this study (see **Figure S1**). All subjects signed a consent form. This project was approved by the Research Ethics Committees of Xinhua Hospital affiliated to Shanghai Jiao Tong University School of Medicine.

### Maternal PFAS Assessment

Blood samples were collected in early pregnancy at a median gestational age of 15 weeks [interquartile range (IQR): 13 – 17 weeks] and centrifuged at 4000 rpm for 10 minutes. Plasma and blood cells were separated and stored at -80 °C until assays. A 100 μl plasma was used for PFAS quantification using high-performance liquid chromatography/tandem mass spectrometry (HPLC/MS-MS; Agilent1290–6490, Agilent Technologies Inc., USA). Detailed methods of PFAS measurement have been described elsewhere (38). Both intra- and inter-assay coefficients of variation (CV) were below 10% for all PFAS congeners. The 10 measured PFAS congeners were PFOS, PFOA, perfluorononanoic acid (PFNA), perfluorodecanoic acid (PFDA), perfluoroundecanoic acid (PFUA), perfluorohexanesulfonate (PFHxS), perfluorododecanoic acid (PFDoA), perfluorobutane sulfonate (PFBS), perfluoroheptanoic acid (PFHpA), perfluorooctane sulfonamide (PFOSA). The limits of detection (LOD) were 0.09 ng/mL for PFOA and PFOS, 0.02 ng/mL for PFDA, PFNA, PFUA and PFHxS, 0.03 ng/mL for PFHpA, 0.05 ng/mL for PFDoA, 0.009 ng/mL for PFBS and 0.12 ng/mL for PFOSA, respectively. PFAS concentrations below the LOD were replaced by the LOD/√2 (39).

### Fetal BDNF and Hg Assessments

The umbilical cord blood samples collected at delivery were kept at room temperature for 30 min for clotting, and then centrifuged at 4000rpm for 10 min. The serum supernatant was then taken and stored at −80°C until testing. Serum BDNF levels were determined using commercially available enzyme-linked immunosorbent assay (ELISA) kit per the manufacturer’s instructions (Human free BDNF Duo Set ELISA Kit; R&D Systems, Minneapolis, MN). All standards and serum samples were analyzed in duplicate, and the average concentration of the duplicate samples was used for the final analyses. The intra- and inter-assay coefficients of variation (CVs) were all below 10%. The logistic curve fitting was used to determine the best fit line and further calculate the corresponding sample concentration. The results are reported in pg/ml.

Our previous study found that the intake of aquatic products (freshwater fish, marine fish, shellfish, shrimp and crab) was positively associated with plasma PFAS concentrations (40). Since fish consumption is associated with mercury exposure (41) and mercury can cause neurotoxicity and a decrease in BDNF level (42), mercury is considered as an important confounder in this study. Serum mercury was determined by cold vapor atomic absorption spectrometry (Model DMA-80; Milestone Inc., Italy). Detailed procedures of the combustion-AAS have been described elsewhere (43). Briefly, after dissolving the whole blood at 4°C temperature, 100μl blood was extracted into the sample cup and injected. Strict quality control measures were implemented to ensure the accuracy of the Hg concentration assessment. The CONTOX (Kaulson Laboratories, Inc., USA) was used as a certified reference material at the beginning and end of every ten sample measurements. An empty sample boat was periodically analyzed. The measured value below the LOD was replaced by LOD/√2.

### Covariates

Data on maternal age, weight, height, education, smoking, alcohol consumption, economic status, child sex and parity were collected using a standardized questionnaire. Pre-pregnant body mass index (BMI) was calculated as weight in kilograms divided by height in meters squared. Maternal education was grouped into high school or below, college or above. In this study, only 2 women (0.3%) were active smokers and 1 woman (0.2%) was regular drinker during pregnancy. Thus, tobacco and alcohol consumption were not included in our final analyses. Parity was classified into nulliparous and parous. Maternal economic status was grouped into four levels based on the question “How do you feel about your family financial situation currently: very good, fairly good, fairly poor and very poor.” Potential confounders were selected based on previous studies and a directed acyclic graph (DAG) (see **Figure S2**).

### Statistical Analysis

We assessed the distribution of all PFAS and relevant covariates. PFAS were expressed as medians and interquartile ranges. Other continuous and categorical covariates were presented as mean and standard deviation, number and percentage, respectively. Before the analyses, all PFAS and Hg concentrations were natural logarithm (ln) transformed due to their skewed distribution characteristics. Differences in PFAS between male and female groups were tested using Wilcoxon’s rank sum test. Spearman rank correlation analysis was conducted to explore the pairwise correlation among PFAS congeners. Multiple imputation with fully conditional specification (FCS-MI) method was used to impute missing values of the covariates before regression analyses. This method can specify the multivariate imputation model on a variable-by-variable basis and offers a principled yet flexible method to address missing data (44). Five datasets with imputations for missing data were created and modeled separately. Results were pooled to obtain the final effect parameters.

We applied multiple linear regression model to assess the associations of PFAS with BDNF level. PFAS congener levels were analyzed one at a time in relation to cord blood BDNF concentration. Tolerance was used to assess the collinearity among covariates. All models adjusted for maternal age, pre-pregnant BMI, maternal education, parity, economic status and Hg level. PFOSA was not included in our regression model due to the low detection rate; all other PFAS had detection rates above 80%. Restricted cubic spline (RCS) models with knots at the 5th, 25th, 50th, 75th and 95th percentiles of each PFAS were fitted to further identify potential non-linearity between plasma PFAS and BDNF level. β-coefficients with *P*-value were used to report the change in BDNF level for every log unit increase in PFAS.

To validate the effects of exposure to multiple PFAS on the outcomes, we further applied a recently developed quantile-based g-computation method to explore the joint effects. This approach combines the inferential simplicity of weighted quantile regression (WQS) with the flexibility of g-computation and allows for nonlinearity and non-additivity of the effects of individual exposures and the mixture as a whole, which appears to be less biased and more robust (45). We used this method to model the joint and individual effects of exposure to all nine PFAS on the BDNF level. Gaussian distribution was specified as link functions. Parameter q was set to 4 as most of the dose-responses did not show a significant nonlinear relationship. 500 bootstrap iterations were performed to calculate the CIs. In addition, stratified analyses were further conducted by child sex.

Several sensitivity analyses were performed to test the robustness of our results. First, in order to assess the representativeness of the study population, we further analyzed the differences in baseline characteristics between the included and the excluded population. Second, PFAS were grouped by tertiles and then included in the multiple linear model one PFAS at a time to explore possible nonlinear relationships. Last, multicollinearity due to high correlation among PFAS congeners may bias the parameter estimates between exposure and outcome. We applied the sparse partial least squares (SPLS) regression to address this issue. This method combines the properties of partial least squares regression (PLS) and least absolute shrinkage and selection operator (LASSO) regression, and has been introduced to assess the health effects of multiple environmental pollutants (46). In the SPLS model, the regression coefficients of PFAS were obtained *via* PLS for the reduced set of PFAS matrix. The number of components (k) and the degree of sparsity (η) are two important parameters that determine the model complexity. We created a series of SPLS models with varied value of k (from 1 to 10 based on the numbers of PFAS) and η (from 0.00 to 0.99 in steps of 0.01). The final combination of k and η was determined by the 10-fold cross-validation with each model running for 100 times and the overall minimum mean squared prediction error. All statistical analyses were conducted in SAS V9.4 (SAS Institute Inc., Cary, NC, USA) and R (version 3.6.1, R Development Core Team 2019). An α level of 5% (two-sided) was considered statistically significant.

## Results

### Population Characteristics

**Table 1** presents the demographic characteristics of the study cohort. The mean maternal age was 28.4±3.6 years. The average pre-pregnant BMI was 21.4±3.1 kg/m^2^. There were slightly more girls than boys. The majority of subjects had college education and were nulliparous. 77.8% of pregnant women had fairly good or very good economic status. The mean Hg exposure level was 2.62±1.63 μg/kg, which differed significantly between male and female fetuses (*P* < 0.001). The average BDNF level was 10797±4713 pg/ml with 9538±5512 and 11025±5416 pg/ml for male and female fetuses, respectively (*P* < 0.001). The maternal age and pre-pregnancy BMI in the excluded population were 28.8±3.7 years and 21.7±3.4 kg/m^2^, respectively, which were slightly higher than which in this study, with *P*<0.05 (**Table S1**). 77% of the excluded women were nulliparous, lower than the included population.

**Table 1:** Demographic characteristics and BDNF levels in the Shanghai Birth Cohort.

Characteristics	N (%)/Mean (SD)
Maternal age (years) [Mean (SD)]	28.4 (3.6)
Pre-pregnant BMI [Mean (SD)]	21.4 (3.1)
Child sex [n (%)]	
Male	347 (48)
Female	369 (51)
Unknown	9 (1)
Maternal educational level [n (%)]	
High school and below	66 (9)
College	654 (90)
Unknown	5 (1)
Parity [n (%)]	
0	608 (84)
1+	98 (13)
Unknown	19 (3)
Economic status [n (%)]	
Very good	99 (14)
Fairly good	465 (64)
Fairly poor	63 (9)
Very poor	7 (1)
Unknown	91 (12)
Hg (μg/kg) [Mean (SD)]	2.62 (1.63)
BDNF (pg/ml) [Mean (SD)]	10797 (4713)

BDNF, brain-derived neurotrophic factor; SD, standard deviation; Hg, mercury.

### Maternal Plasma PFAS and Hg

**Table 2** describes the LOD and detection rate, the median and interquartile range (IQR) of Hg and each PFAS congener. The detection rate of Hg exceeded 99% and the median concentration was 2.32 μg/kg. PFOSA had a low detection rate (23%) and, therefore, was not analyzed further. PFOA and PFOS were the dominant compounds with the median concentration at 11.63 and 9.69 ng/ml, respectively. The concentrations of short-chain PFAS like PFBS and PFHpA were relatively low (0.04 and 0.06 ng/ml, respectively). PFDA concentrations differed significantly between male and female fetuses. Pairwise correlation analysis shows that there were strong correlations among the long-chain PFAS (PFOS, PFNA, PFDA and PFUA) with r ≥ 0.6 (**Figure S3**).

**Table 2:** Perfluoroalkyl substances (PFAS, ng/mL) exposure level during early pregnancy, stratified by child sex.

Exposure items	All subjects (N=725)			Male (N=347)	Female (N=369)	*P*-value
	LOD	<LOD (%)	Median (IQR)	Median (IQR)	Median (IQR)	
Hg	0.15	0.76	2.32 (1.59)	2.44 (1.64)	2.17 (1.41)	0.01
PFOA	0.09	0.00	11.52 (6.02)	11.63 (6.44)	11.50 (5.57)	0.31
PFOS	0.09	0.00	9.36 (6.97)	9.69 (7.23)	8.99 (6.90)	0.13
PFNA	0.02	0.00	1.68 (1.11)	1.74 (1.11)	1.60 (1.14)	0.11
PFDA	0.02	0.00	1.65 (1.35)	1.73 (1.40)	1.56 (1.27)	0.03
PFUA	0.02	0.00	1.39 (1.04)	1.44 (1.04)	1.34 (1.02)	0.05
PFHxS	0.02	0.00	0.17 (0.14)	0.18 (0.14)	0.16 (0.15)	0.49
PFDoA	0.05	12.01	0.50 (0.26)	0.51 (0.25)	0.50 (0.26)	0.52
PFBS	0.009	12.54	0.04 (0.04)	0.04 (0.04)	0.04 (0.03)	0.78
PFHpA	0.03	18.48	0.06 (0.06)	0.06 (0.06)	0.06 (0.07)	0.62
PFOSA	0.12	76.55	0.08 (0.14)	0.08 (0.14)	0.08 (0.14)	0.17

LOD, the limit of detection; IQR, interquartile range; PFOA, perfluorooctanate; PFOS, perfluorooctane sulfonate; PFNA, perfluorononanoic acid; PFDA, perfluorodecanoic acid; PFUA, perfluoroundecanoic acid; PFHxS, perfluorohexanesulfonate; PFDoA, perfluorododecanoic acid; PFBS, perfluorobutane sulfonate; PFHpA, perfluoroheptanoic acid; PFOSA, perfluorooctane sulfonamide.

### Single Pollutant Analyses of PFAS and Blood BDNF Level

**Table 3** shows the associations between single PFAS and BDNF level in a multiple linear model. A significant positive association was observed between PFHxS and BDNF level after adjusting for potential confounders (β=1285, 95% CI: 453, 2118, *P*=0.003). Other PFAS congeners but PFDoA showed positive trends. However, all these trends were statistically non-significant.

**Table 3:** Associations of log-transformed PFAS concentration during pregnancy with BDNF levels in cord blood^*^.

Exposure items	β (95%CI)^a^	*P*	β (95%CI)^b^	*P*
PFOA	253 (-606, 1112)	0.56	236 (-617, 1088)	0.59
PFOS	176 (-452, 804)	0.58	269 (-374, 911)	0.41
PFNA	55 (-604, 715)	0.87	203 (-474, 879)	0.56
PFDA	-31 (-594, 533)	0.92	81 (-499, 660)	0.79
PFUA	2 (-588, 592)	0.99	73 (-535, 681)	0.81
PFHxS	1287 (444, 2130)	0.003	1285 (453, 2118)	0.003
PFDoA	-191 (-717, 497)	0.72	-130 (-759, 500)	0.69
PFBS	169 (-335, 672)	0.51	148 (-364, 660)	0.57
PFHpA	220 (-200, 641)	0.3	233 (-180, 645)	0.27

^*^Multiple linear regression was used to estimate the linear associations. Effect change was expressed as Beta coefficient (β), 95% confident interval (95%CI) and p-value (P).

a No adjustment for any covariates.

b Adjusted for maternal age, pre-pregnant BMI, maternal education, parity, child sex, economic status and Hg.

### Mixture Analyses of PFAS With BDNF

**Table 4** presents the results of quantile-based g-computation analyses for PFAS mixture and BDNF level. As for the total subjects, prenatal PFAS exposure and BDNF levels had the same trend, but the association was not significant (β=447, 95% CI: -83, 978, *P*=0.10) for every quantile increase in PFAS exposure levels. PFHxS still showed significantly positive association with BDNF level after adjusting for covariates and other PFAS congeners (β=592, 95% CI: 226, 958, *P*=0.002). In addition, PFOA was found to be negatively associated with BDNF level (β=-520, 95% CI: -942, -98, *P*=0.02).

**Table 4:** The total effect of exposure mixture and independent effect of individual PFAS after controlling for other exposure items on BNDF levels in cord blood^*^.

Items	β (95%CI)^a^	*P*	β (95%CI)^b^	*P*
All subjects (N=725)				
PFAS mixture	405 (-114, 924)	0.13	448 (-83, 978)	0.10
PFOA	-443 (-863, -22)	0.04	-520 (-942, -98)	0.02
PFOS	141 (-438, 720)	0.63	141 (-436, 718)	0.63
PFNA	319 (-312, 950)	0.32	399 (-233, 1031)	0.22
PFDA	-200 (-979, 579)	0.62	-123 (-894, 647)	0.75
PFDA	-148 (-842, 546)	0.68	-164 (-861, 532)	0.64
PFHxs	606 (236, 977)	0.001	592 (226, 958)	0.002
PFDoA	-197 (-591, 198)	0.33	-244 (-639, 151)	0.23
PFBS	42 (-299, 383)	0.81	75 (-269, 420)	0.67
PFHpA	284 (-71, 639)	0.12	291 (-59, 641)	0.10
Male (N=347)				
PFAS mixture	771 (6, 1537)	0.05	773 (25, 1520)	0.04
PFOA	-495 (-1052, 62)	0.08	-553 (-1129, 23)	0.06
PFOS	163 (-596, 923)	0.67	152 (-623, 928)	0.70
PFNA	311 (-495, 1117)	0.45	317 (-516, 1149)	0.46
PFDA	-181 (-1137, 775)	0.71	-52 (-1038, 934)	0.92
PFDA	-57 (-927, 813)	0.90	-109 (-1016, 799)	0.81
PFHxS	751 (244, 1258)	0.004	757 (239, 1275)	0.004
PFDoA	-36 (-589, 516)	0.90	-67 (-640, 507)	0.82
PFBS	277 (-330, 884)	0.35	297 (-339, 933)	0.34
PFHpA	38 (-456, 533)	0.88	30 (-486, 545)	0.91
Female (N=369)				
PFAS mixture	137 (-656, 931)	0.73	105 (-792, 1002)	0.82
PFOA	-171 (-780, 437)	0.58	-281 (-916, 354)	0.39
PFOS	-263 (-1130, 604)	0.55	-277 (-1160, 606)	0.54
PFNA	248 (-635, 1131)	0.58	416 (-487, 1319)	0.37
PFDA	-438 (-1558, 683)	0.44	-351 (-1485, 783)	0.54
PFDA	489 (-519, 1497)	0.34	271 (-755, 1296)	0.60
PFHxS	483 (-52, 1017)	0.08	522 (-16, 1061)	0.06
PFDoA	-451 (-1016, 115)	0.12	-473 (-1034, 89)	0.10
PFBS	-219 (-762, 325)	0.43	-223 (-800, 353)	0.44
PFHpA	460 (-63, 984)	0.08	501 (-20, 1021)	0.06

^*^Quantile-based g-computation was used to estimate the mixed and independent effects of PFAS. Effect change was expressed as Beta coefficient (β), 95% confident interval (95%CI) and p-value (P). All exposure variables were log-transformed.

^a^Mixed and independent effects of 9 PFAS congeners on BDNF level without adjusting for any covariate.

^b^Mixed and independent effects of 9 PFAS congeners on BDNF level, adjusting for maternal age, pre-pregnant BMI, maternal education, parity, child sex, economic status and Hg.

As for the stratified analyses by fetal sex, the mixed effect of PFAS on BDNF level in male fetuses was significant (β=773, 95% CI: 25, 1520, *P*=0.04). PFHxS was the main culprit. For female fetuses, PFAS did not significantly affect BDNF level. In general, results of the stratified analyses indicated that the joint effects of nine PFAS on BDNF level were more pronounced in male fetuses.

### Sensitivity Analyses

**Table S2** shows the results of multiple linear regression with PFAS included as an ordinal variable. Overall, the results were consistent with **Table 3**. The middle and highest tertiles of PFHxS exposure were positively associated with increased BDNF level [β=875 (95% CI: 53, 1697, *P*=0.04) for T2 and β=1072 (95% CI: 246, 1898, *P*=0.01) for T3, respectively]. In male fetuses, PFHxS, but not other congeners, was statistically correlated with the BDNF level [β=1142 (95% CI: 25, 2259, *P*=0.05) for T2 and β=1445 (95% CI: 324, 2567, *P*=0.01) for T3, respectively].

To address the concern of the high correlations among some PFAS congeners and validate the results of mixed exposure models, SPLS was used. Only PFHxS and fetal sex were finally selected by the SPLS model, which was consistent with the results of the multiple linear regression and the quantile-based g-computation (**Table S3**). Results of RCS show that non-linear association was not found between PFAS exposure and BDNF level except for that of PFOA exposure (*P*=0.020) (**Figure S3**).

## Discussion

Our large prospective cohort found that PFHxS exposure was significantly associated with an increased BDNF level. In general, no significant association between PFAS mixture and BDNF level was found due to the bidirectional effects of different PFAS congeners. The association between PFAS and BDNF levels seem to be more pronounced in male fetuses.

Although the PFDA plasma concentration was sex-dimorphic in our study, there was no significant association with BDNF level. Only one previous study found a weak positive correlation between PFDA exposure and the total SDQ score (18). The HOME study showed that higher concurrent concentrations of PFHxS were associated with shorter (better) times. Higher prenatal PFHxS was positively associated with percentage of traveling distance in the correct quadrant, indicating better performance, which is consistent with our result (22). However, another study showed that prenatal and childhood PFHxS were not associated with WISC-IV measures (9). Similar null association was reported by a study in the Faroe Islands birth cohort (18). Although results of the MoBa cohort found a negative association between PFHxS prenatal exposure and nonverbal working memory, the relationship was weak and unclear (47). Result from the INMA Project showed a negative pattern between PFHxS and 14-month motor development, which needs to be verified in more population-based studies (48).

A recent *in vitro* study found that PFHxS exposure produced morphometric effects in the zebrafish larvae, specifically increased body length, and was associated with elevated BDNF levels (49). As for the above inverse or null associations for PFAS exposure and BDNF, it is biologically plausible that the protective associations between early-pregnancy PFAS exposure and neurodevelopment was observed, because previous *in vitro* studies reported that PFOA and PFOS were agonists of peroxisome proliferator-activated receptors-γ (PPAR-γ) (50, 51). The activation of this receptor may be neuroprotective (52). PPAR-γ shows a relatively low level of expression primarily limited to the granule cells of the hippocampal dentate gyrus (52). Some PPAR-γ are expressed in the caudate putamen and globus pallidus of the basal ganglia, thalamus and the piriform cortex (53). Studies indicate that PPAR-γ expression in brain is mostly localized in the microglia and astrocytes, which is also the main area of BNDF expression, and the cell types could play a significant role in the inflammatory responses of the central nervous system (54).

Another possible explanation for the negative and null correlation is that there may be other toxic pathways at work, especially the possible interferrence of the thyroid by PFAS during pregnancy. Animal Studies showed that PFAS may affect neuronal plasticity through increasing levels of the proteins synaptophysin, CaMKII, GAP-43 and Tau, all of which are involved in neuronal growth and synaptogenesis (55). PFAS can involve thyroxine (T4) for binding to transthyretin, increase thyroid-stimulating hormone (TSH) and decrease free thyroxine (fT4), all of which can transfer from mother to fetus and are critical for normal brain development in early pregnancy (56, 57). Results of *in vitro* studies indicated that the detrimental effects of PFAS on thyroid cells would include the bioaccumulation, cytotoxicity and genotoxicity.

PFAS interfere TH synthesis, affecting TPO function and iodine uptake. PFAS exposure during pregnancy seems to cause slight but statistically significant alterations in thyroid function parameters, both in mothers and newborns (58). Both legacy PFAS and some new PFAS alternatives that were not included in our PFAS detection profiles such as hexafluoropropylene oxide dimer acid (HFPO-DA or GenX) and 6:2 chlorinated polyfluorinated ether sulfonate (F-53B) display a highly toxic profile on thyroid cells through promoting DNA-damage and affecting the expression of thyroid transcription-factor genes (59, 60). Other short-chain PFAS alternatives such as PFBS and perfluorobutanoic acid (PFBA) seemed to have little effect on thyroid cell function and viability (61). Therefore, the elevated BDNF levels associated with PFHxS exposure may indicate better neurobehavioral development outcomes, but other unconsidered PFAS, especially the neurotoxic and endocrine disrupting effect of new generation PFAS alternatives, should not be simply dismissed. More studies are waranted to clarify the mechanism of PFAS-induced neurotoxicity and whether BDNF plays a role in it.

Male fetuses appeared to be more sensitive to PFAS-induced BDNF changes. A study from the World Trade Center (WTC) birth cohort found that child sex could modify the association between PFOS and the mental development index, with the observed relationship being positive for females and negative for males (19). The C8 Health Study found PFAS levels in children aged 2 to 8 years to be related to improved executive function among boys (62). In contrast, studies in Shanghai-Minhang Birth Cohort reported that PFAS concentrations tended to be negatively associated with increased risk of development problem in girls at 4 years of age (14). Girls had consistently positive associations between PFAS exposure and the Strengths and Difficulties Questionnaire (SDQ) scores (20). Yet, the Viva Project did not find a consistent pattern of effect measure modification by child sex in the association of PFAS and child cognition (8). Mercury exposure was another important pollutant and also has gender differences in its effect, which may be related to the enzyme levels regulating mercury metabolism in different genders and the suppression of mercury-induced oxidative stress *via* hormonal effects in female offspring (63). More studies are needed to confirm the possible sex dimorphic pattern of PFAS induced neurobehavior and relevant molecular changes.

Besides, a number of studies have reported positive links between some PFAS isomer exposure and impaired neurodevelopment. For example, a birth cohort study in Taiwan found that prenatal exposure to PFOS affected the gross motor development domain at 2 years of age (7). A population-based cohort study in the U.S. found that a high PFOA exposure level was associated with an elevated risk of hypotonic phenotypes in young infant, while infants with hypotonic phenotypes had lower psychomotor development and lower externalizing scores at 2–3 years of age (64, 65). This study also found that prenatal exposure to PFOS, but not PFOA, was associated with decreased executive functions in children aged at 5 and 8 years (66). However, there are still many reports of negative or null association. The Denmark Birth Cohort (DNBC) was the largest study that found no apparent associations between prenatal PFOS and PFOA concentrations and development milestones, standardized written exam score, depression or childhood autism (67, 68). Results from the C8 Health Study found that geospatially estimated intrauterine PFOA exposure was associated with higher full-scale IQ and no apparent associations were found with childhood math skills, language, memory and learning and visual spatial processing (69). Similar null association was found in the Norwegian birth cohort (20).

Several reasons may explain previous inconsistent findings. First, the exposure levels, ranges, sources and PFAS mixture composition differed among study regions and populations. Second, the study design and statistical model applied in various studies were quite different. Differences in neurobehavioral assessment instruments may be another important source of heterogeneity. Third, the potential confounding effects of other environmental pollutants are always a challenge for the exposure-outcome causal inference. Lastly, the mechanism that affects neurobehavioral development is very complex, but the sample size of previous studies was generally small, and may not have had enough statistical power to detect subtle differences.

In addition, previous studies on the associations between environmental contaminants and neurodevelopment were usually cross-sectional in design and the contaminants were evaluated one by one, which may introduce bias by reverse causation and miss the correlations between pollutants (70). This calls for a mixed exposure analysis to assess the effect of PFAS mixture and Hg exposure. Our study used a novel quantile-based g-computation to assess the mixed effects, which allows estimation of parsimonious mixture dose–response parameters by a joint marginal structural model (45). The mixed exposure analysis also took into account the non-linear associations between pollutants and health outcomes and helped to better assess the overall health effects of specific types of environmental pollutants. In the current study, the PFAS isomers except for PFOA did not show a significant non-linear relationship. Results of the multiple linear regression after treating PFOA as categorical variable did not suggest a significant association with BDNF level. Therefore, we used qgcomp model to conduct mixed effect analyses based on the linear assumption, which has a smaller bias in the parameter estimation.

Our study has several strengths. First, to our best knowledge, this is the first study to explore the associations between prenatal PFAS exposure and fetal BDNF level, which helps to better understand the neurotoxic mechanism of PFAS. Second, our study was a prospective cohort study with a clear temporal relationship between exposure and outcome. Third, we employed a quantile-based g-computation method to investigate the effects of mixture exposure and control the possible confounding effects of Hg exposure. Compared with traditional single-pollutant model, these methods have higher flexibility and smaller error bias.

Still, our study has some limitations. First, although we have adjusted for as many potential confounders as possible, other important factors such as endocrine disruptors (phenols, phthalate, polybrominated diphenyl ethers), lead, manganese and cadmium were not included. We could not completely rule out the possibility of residual confounding. Second, only a single estimate of plasma PFAS concentrations in early pregnancy was determined in our study. The timing of exposure can play an important role in the toxicity of PFAS given the different developmental timing for organ systems. Though correlations for PFOA and PFOS measures between early- and late-pregnancy serum samples were found to be high (71), other physiological factors such as maternal blood volume expansion and metabolic changes during pregnancy could add uncertainty to the exposure measures (72). Moreover, the transplacental transfer from the mother to the fetuses might vary by chemicals (73). All of these factors can result in exposure misclassification or measurement errors in fetal exposure level. If the measurement errors are random and non-differential, effect estimates might be biased towards the null. Last, although BDNF has been thoroughly studied as a marker of brain development during gestation, it represents a rather indirect marker of neurodevelopment. No data regarding neurological performance of newborns are included in this study. Future research should pay more attention to the direct or indirect relationships between PFAS exposure, BDNF level and neurobehavioral development outcomes.

## Conclusions

Our study found that prenatal exposure to PFHxS was associated with increased BDNF level. No association of other PFAS congeners and Hg exposure with BDNF and no mixed effects were found. The effect of PFHxS on BDNF during pregnancy seemed more pronounced in male fetuses.

## Data Availability Statement

The raw data supporting the conclusions of this article will be made available by the authors, without undue reservation.

## Ethics Statement

The studies involving human participants were reviewed and approved by Ethics Committee of Xinhua Hospital Affiliated to Shanghai Jiaotong University School of Medicine. The patients/participants provided their written informed consent to participate in this study. Written informed consent was obtained from the individual(s) for the publication of any potentially identifiable images or data included in this article.

## Author Contributions

GY and FL did the background research, collected, and matched the data. FL, MN, SL, and BL participated in the pollutant and BDNF detection, and take responsibility for the integrity and accuracy of the exposure data. GY and FL analyzed and interpreted the data, and drafted the manuscript. JZ is the designer of SBC, and had full access to all the delivery data in the study. LF and JZ supervised the project and carefully revised the manuscript. Each author contributed important intellectual content during manuscript drafting or revision. All authors accept accountability for the overall work. All authors contributed to the article and approved the submitted version.

## Funding

This study was funded by the National Natural Science Foundation of China (41991314 and 81530086) and the Collaborative Innovation Program of the Shanghai Municipal Health Commission (2020CXJQ01), the Ministry of Science and Technology of China (2019YFA0802501).

## Conflict of Interest

The authors declare that the research was conducted in the absence of any commercial or financial relationships that could be construed as a potential conflict of interest.
