# Supplementary material for: Exposure to Perfluoroalkyl Substances During Pregnancy and Fetal BDNF Level: A Prospective Cohort Study
Source: Front Endocrinol (Lausanne). 2021 Jun 1;12:653095. doi: 10.3389/fendo.2021.653095 (PMC8204808; doi:10.3389/fendo.2021.653095)
Supplement: Supplementary file 1 [file DataSheet_1.docx]

**Table S1**

**Comparative analysis of the basic demographic characteristics between the included and excluded populations.**

| **Characteristics** | **N (%)/Mean (SD) N=725** | **N (%)/Mean (SD)**  **N=3456** | ***p*** |
| --- | --- | --- | --- |
| **Maternal age (years) [Mean (SD)]** | 28.4 (3.6) | 28.8 (3.7) | 0.003 |
| **Pre-pregnant BMI [Mean (SD)]** | 21.4 (3.1) | 21.7 (3.4) | 0.025 |
| **Child sex [n (%)]** |  |  | 0.067 |
| Male | 347 (48) | 1569(45) |  |
| Female | 369 (51) | 1716(50) |  |
| Unknown | 9 (1) | 171(5) |  |
| **Maternal educational level [n (%)]** |  |  | 0.582 |
| High school and below | 66 (9) | 294(8) |  |
| College | 654 (90) | 2693(78) |  |
| Unknown | 5 (1) | 469(14) |  |
| **Parity [n (%)]** |  |  | <.0001 |
| 0 | 608 (84) | 2659(77) |  |
| 1+ | 98 (13) | 686(20) |  |
| Unknown | 19 (3) | 111(3) |  |
| **Economic status [n (%)]** |  |  | 0.471 |
| Very good | 99 (14) | 430(12) |  |
| Fairly good | 465 (64) | 1580(46) |  |
| Fairly poor | 63 (9) | 262(8) |  |
| Very poor | 7 (1) | 26(1) |  |
| Unknown | 91 (12) | 1158(33) |  |

**Table S2**

**Associations of PFAS concentration in early pregnancy with BDNF level in cord blood^*^.**

| PFASs | All subjects  [β (*P*)^a^] |  | All subjects  [β (*P*)^b^] |  | Male subjects  [β (*P*)^b^] |  | Female subjects  [β (*P*)^b^] |  |
| --- | --- | --- | --- | --- | --- | --- | --- | --- |
| PFOA |  |  |  |  |  |  |  |  |
| T1 | 1.00 (Ref) |  | 1.00 (Ref) |  | 1.00 (Ref) |  | 1.00 (Ref) |  |
| T2 | -353 (-1192, 487) | 0.41 | -460 (-1287, 368) | 0.28 | -42 (-1190, 1106) | 0.94 | -644 (-1860, 572) | 0.30 |
| T3 | 30 (-810, 869) | 0.94 | -7 (-839, 824) | 0.99 | 210 (-904, 1324) | 0.71 | -66 (-1335, 1204) | 0.92 |
| PFOS |  |  |  |  |  |  |  |  |
| T1 | 1.00 (Ref) |  | 1.00 (Ref) |  | 1.00 (Ref) |  | 1.00 (Ref) |  |
| T2 | -193 (-1032, 646) | 0.65 | -180 (-1014, 653) | 0.67 | 185 (-952, 1323) | 0.75 | -455 (-1664, 753) | 0.46 |
| T3 | 395 (-444, 1234) | 0.36 | 462 (-391, 1314) | 0.29 | 864 (-284, 2012) | 0.14 | 159 (-1104, 1421) | 0.81 |
| PFNA |  |  |  |  |  |  |  |  |
| T1 | 1.00 (Ref) |  | 1.00 (Ref) |  | 1.00 (Ref) |  | 1.00 (Ref) |  |
| T2 | -602 (-1440, 236) | 0.16 | -542 (-1378, 293) | 0.2 | -105 (-1258, 1049) | 0.86 | -960 (-2175, 254) | 0.12 |
| T3 | 234 (-604, 1072) | 0.58 | 386 (-463, 1234) | 0.37 | 937 (-224, 2098) | 0.11 | -59 (-1292, 1174) | 0.93 |
| PFDA |  |  |  |  |  |  |  |  |
| T1 | 1.00 (Ref) |  | 1.00 (Ref) |  | 1.00 (Ref) |  | 1.00 (Ref) |  |
| T2 | -107 (-947, 732) | 0.16 | -71 (-911, 770) | 0.20 | 482 (-673, 1637) | 0.86 | -621 (-1832, 589) | 0.12 |
| T3 | 231 (-608, 1071) | 0.58 | 388 (-466, 1242) | 0.37 | 772 (-378, 1923) | 0.11 | 84 (-1186, 1353) | 0.93 |
| PFUA |  |  |  |  |  |  |  |  |
| T1 | 1.00 (Ref) |  | 1.00 (Ref) |  | 1.00 (Ref) |  | 1.00 (Ref) |  |
| T2 | 435 (-405, 1274) | 0.31 | 440.71 (-398.47, 1279.90) | 0.3 | 951 (-203, 2105) | 0.11 | 14 (-1194, 1222) | 0.98 |
| T3 | 132 (-708, 971) | 0.76 | 250.22 (-605.61, 1106.04) | 0.57 | 832 (-320, 1985) | 0.16 | -264 (-1533, 1005) | 0.68 |
| PFHxS |  |  |  |  |  |  |  |  |
| T1 | 1.00 (Ref) |  | 1.00 (Ref) |  | 1.00 (Ref) |  | 1.00 (Ref) |  |
| T2 | 903 (67, 1738) | 0.03 | 875 (53, 1697) | 0.04 | 1142 (24, 2259) | 0.05 | 649 (-564, 1861) | 0.29 |
| T3 | 1081 (245, 1917) | 0.01 | 1072 (246, 1898) | 0.01 | 1445 (324, 2567) | 0.01 | 850 (-373, 2074) | 0.17 |
| PFDoA |  |  |  |  |  |  |  |  |
| T1 | 1.00 (Ref) |  | 1.00 (Ref) |  | 1.00 (Ref) |  | 1.00 (Ref) |  |
| T2 | -322 (-1237, 592) | 0.49 | -317 (-1251, 617.17) | 0.50 | 509 (-891, 1910) | 0.47 | -1064 (-2274, 146) | 0.08 |
| T3 | -82 (-974, 810) | 0.86 | -101 (-1011, 809.43) | 0.83 | 645 (-579, 1870) | 0.30 | -777 (-2050, 496) | 0.23 |
| PFBS |  |  |  |  |  |  |  |  |
| T1 | 1.00 (Ref) |  | 1.00 (Ref) |  | 1.00 (Ref) |  | 1.00 (Ref) |  |
| T2 | 147 (-770, 1064) | 0.75 | 125 (-747, 996) | 0.78 | 294 (-916, 1505) | 0.63 | -48 (-1374, 1279) | 0.94 |
| T3 | 129 (-792, 1050) | 0.78 | 201 (-739, 1140) | 0.67 | 838 (-783, 2459) | 0.29 | -465 (-1805, 875) | 0.49 |
| PFHpA |  |  |  |  |  |  |  |  |
| T1 | 1.00 (Ref) |  | 1.00 (Ref) |  | 1.00 (Ref) |  | 1.00 (Ref) |  |
| T2 | -334 (-1173, 505) | 0.44 | -263 (-1089, 563) | 0.53 | -442 (-1577, 694) | 0.45 | -68 (-1271, 1136) | 0.91 |
| T3 | 179 (-660, 1018) | 0.68 | 230 (-596, 1055) | 0.59 | -106 (-1230, 1017) | 0.85 | 547 (-671, 1766) | 0.38 |

^*^ Multiple linear regression was adopted to estimate the linear associations. Effect change was expressed as Beta coefficient (β), 95% confident interval (95%CI) and *p*-value (*P*). PFAS and Hg are classified into three different levels (T1, T2, T3) according to their tertiles, respectively.

^a^ No adjustment for any covariates.

^b^ Adjusted for maternal age, pre-pregnant BMI, maternal education, parity, child sex, economic status and Hg.

**Table S3**

**Estimates of association between PFAS, Hg and BDNF in cord blood by sparse partial least squares (SPLS).**

|  | Imputation=1, coef | Imputation=2, coef | Imputation=3, coef | Imputation=4, coef | Imputation=5, coef |
| --- | --- | --- | --- | --- | --- |
| Var | η=0.99, k=3 | η=0.99, k=3 | η=0.92, k=3 | η=0.99, k=2 | η=0.99, k=2 |
| Maternal age | 0 | 0 | 0 | 0 | 0 |
| Pre-pregnant BMI | 0 | 0 | 0 | 0 | 0 |
| Parity | -372.93 | -349.80 | 0 | 0 | 0 |
| Maternal education | 0 | 0 | 0 | 0 | 0 |
| Child sex | 891.18 | 891.04 | 919.07 | 859.89 | 851.97 |
| Economic status |  |  |  |  |  |
| Fairly good | 0 | 0 | 0 | 0 | 0 |
| Fairly poor | 0 | 0 | 0 | 0 | 0 |
| Very poor | 0 | 0 | 0 | 0 | 0 |
| Hg | 0 | 0 | 0 | 0 | 0 |
| PFOA | 0 | 0 | 0 | 0 | 0 |
| PFOS | 0 | 0 | 0 | 0 | 0 |
| PFNA | 0 | 0 | 0 | 0 | 0 |
| PFDA | 0 | 0 | 0 | 0 | 0 |
| PFDA | 0 | 0 | 0 | 0 | 0 |
| PFHxS | 552.38 | 545.69 | 548.51 | 550.60 | 552.61 |
| PFDoA | 0 | 0 | 0 | 0 | 0 |
| PFBS | 0 | 0 | 0 | 0 | 0 |
| PFHpA | 0 | 0 | 0 | 0 | 0 |

^a^ Imputation=1~Imputation=5 refer to different imputed datasets. Coef: the Regression coefficient calculated using SPLS models; 0 indicates association was not selected in SPLS model; SPLS tuning parameters: η: the degree of sparsity; k: the number of components used to construct the model.


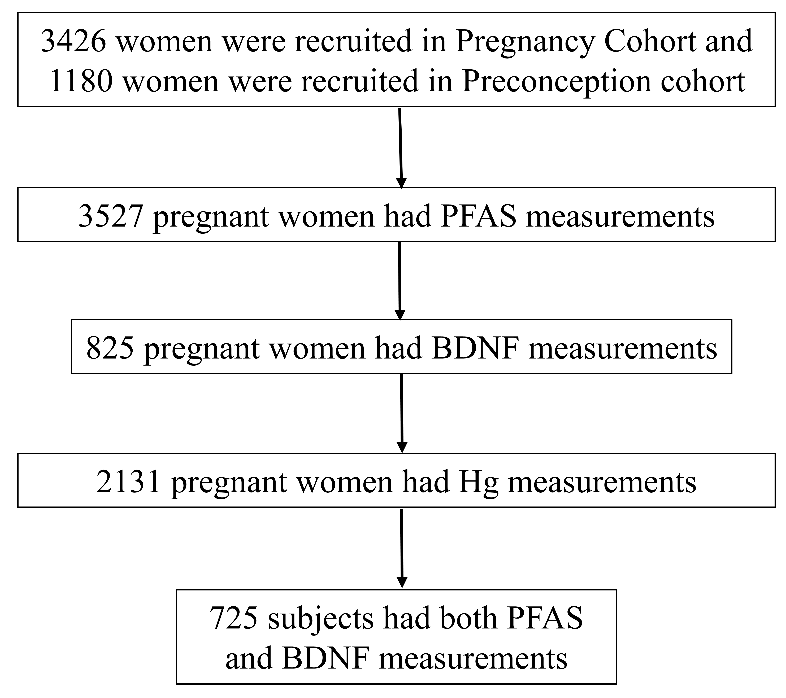


**Fig S1. The flow chart for sample size inclusion criteria.**


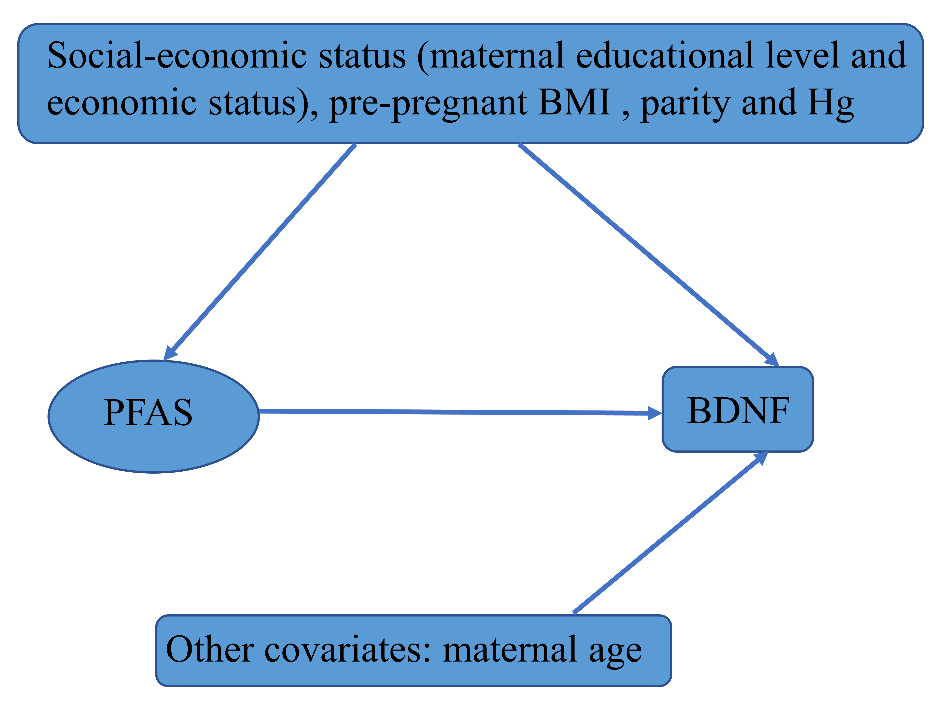


**Fig S2. Directed Acyclic Graph for covariate selection.**


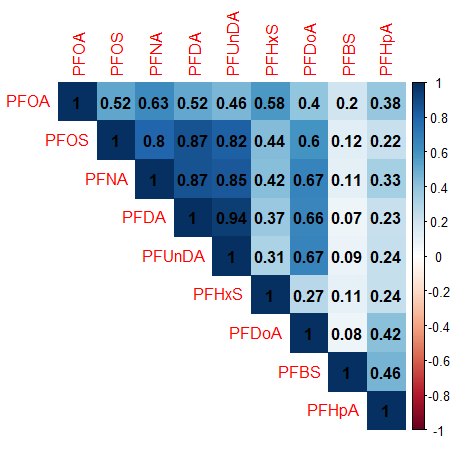


**Fig S3. Pairwise correlation between plasma concentrations of different PFAS congeners in early pregnancy.**


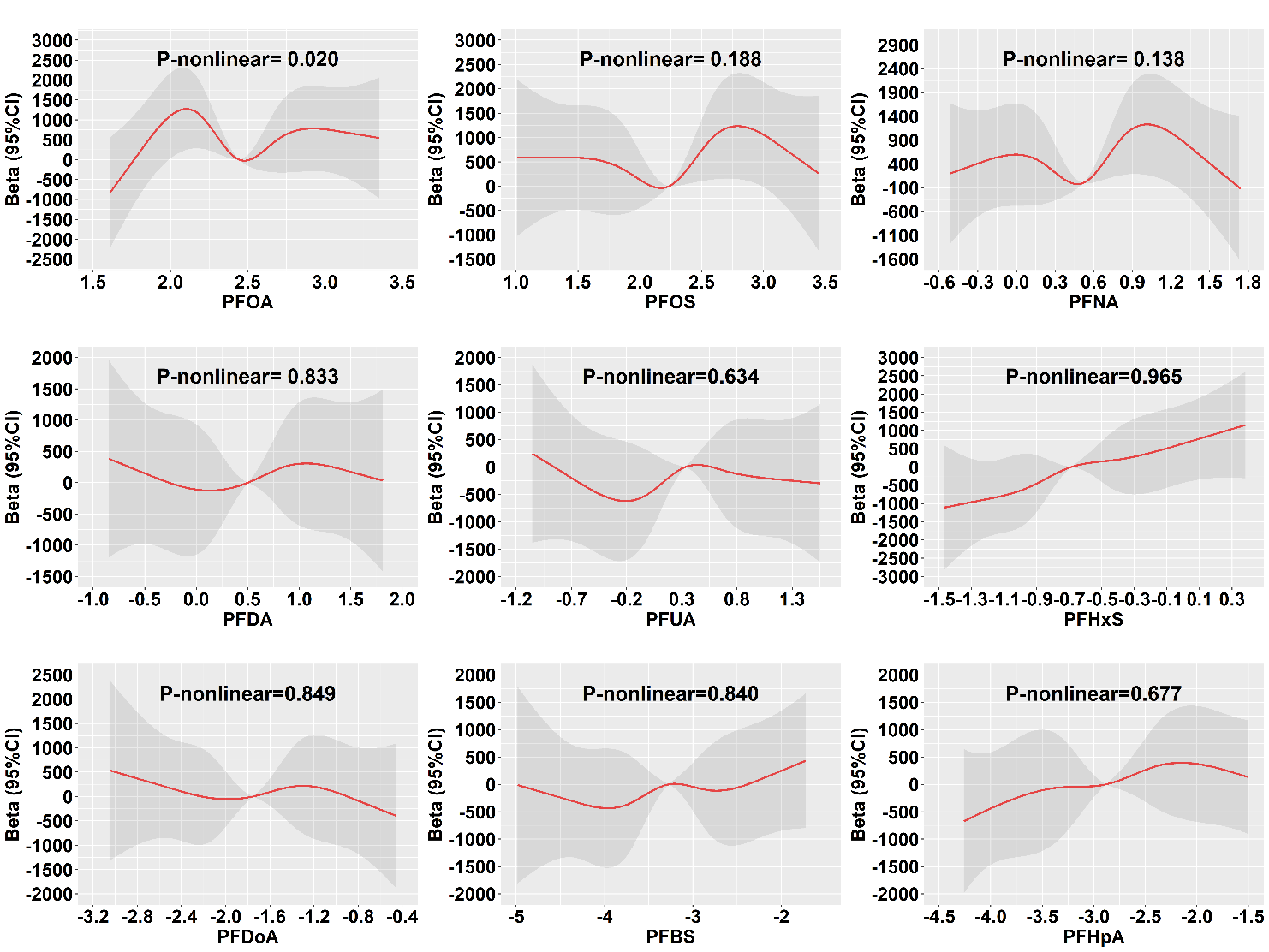


**Fig S4. Non-linear associations between each of the log-transformed PFAS and BDNF level. Solid lines (red) correspond to the beta coefficient, and shaded regions correspond to the 95% confidence intervals of the beta coefficient. Knots were fixed at the 5th, 25th, 50th, 75th and 95th percentiles of the log-transformed PFAS.**
